# Effects of Osmotic Stress on the mRNA Expression of *prl*, *prlr*, *gr*, *gh*, and *ghr* in the Pituitary and Osmoregulatory Organs of Black Porgy, *Acanthopagrus schlegelii*

**DOI:** 10.3390/ijms24065318

**Published:** 2023-03-10

**Authors:** Ganesan Nagarajan, Adimoolam Aruna, Yu-Ming Chang, Yousef Ahmed Alkhamis, Roshmon Thomas Mathew, Ching-Fong Chang

**Affiliations:** 1 Department of Basic Sciences, PYD, King Faisal University, Al Ahsa 31982, Saudi Arabia; 2 Center of Excellence for the Oceans, National Taiwan Ocean University, Keelung 20224, Taiwan; 3 Department of Aquaculture, National Taiwan Ocean University, Keelung 20224, Taiwan; 4 Animal and Fish Production Department, College of Agricultural and Food Sciences, King Faisal University, Hofuf-420, Al-Asha 31982, Saudi Arabia; 5 Fish Resources Research Center, King Faisal University, Hofuf-420, Al-Asha 31982, Saudi Arabia

**Keywords:** pituitary hormone, *prlr*, *gr*, *ghr*, *α-nka*, osmoregulation, fish

## Abstract

In euryhaline teleost black porgy, *Acanthopagrus schlegelii*, the glucocorticoid receptor (*gr*), growth hormone receptor (*ghr*), prolactin (*prl*)-receptor (*prlr*), and sodium–potassium ATPase alpha subunit (*α-nka*) play essential physiological roles in the osmoregulatory organs, including the gill, kidney, and intestine, during osmotic stress. The present study aimed to investigate the impact of pituitary hormones and hormone receptors in the osmoregulatory organs during the transfer from freshwater (FW) to 4 ppt and seawater (SW) and vice versa in black porgy. Quantitative real-time PCR (Q-PCR) was carried out to analyze the transcript levels during salinity and osmoregulatory stress. Increased salinity resulted in decreased transcripts of *prl* in the pituitary, *α-nka* and *prlr* in the gill, and *α-nka* and *prlr* in the kidney. Increased salinity caused the increased transcripts of *gr* in the gill and *α-nka* in the intestine. Decreased salinity resulted in increased pituitary *prl*, and increases in *α-nka* and *prlr* in the gill, and *α-nka*, *prlr*, and *ghr* in the kidney. Taken together, the present results highlight the involvement of *prl*, *prlr*, *gh*, and *ghr* in the osmoregulation and osmotic stress in the osmoregulatory organs (gill, intestine, and kidney). Pituitary *prl*, and gill and intestine *prlr* are consistently downregulated during the increased salinity stress and vice versa. It is suggested that *prl* plays a more significant role in osmoregulation than *gh* in the euryhaline black porgy. Furthermore, the present results highlighted that the gill *gr* transcript’s role was solely to balance the homeostasis in the black porgy during salinity stress.

## 1. Introduction

The end product of the hypothalamic–pituitary–interrenal (HPI) axis, “cortisol”, is a key endogenous glucocorticoid that has versatile function such as in respiration, osmoregulation, reproduction, immune responses, growth, and metabolism in teleosts [1]. It is well-known that during stress, cortisol forms a complex with the ligand-activated transcriptional factor, glucocorticoid receptor (*gr*), to bind specifically to the glucocorticoid response elements (GREs), which increases the transcription of target genes and takes a role in energy metabolism, inflammation, and neuromodulation [2,3,4]. In teleosts, there are two forms of *gr* genes (*gr1* and *gr2*), and one mineralocorticoid receptor (*mr*) gene has been identified [5,6]. The trout *gr1* had an additional nine amino acid residues (WRARQNTDG) in the DNA-binding domain [7]. An additional nine residues in *gr1* confer a stronger binding affinity of the receptor to the GRE-responsive promoter, which corresponds with higher constitutive transcriptional activity [8]. In fish, cortisol stimulation and salinity stress both increase the amount of *gr* mRNA in different osmoregulatory tissues [9,10,11,12,13]. *gr* facilitates seawater (SW) acclimation by stimulating the proliferation and differentiation of ion-transporting chloride cells in the gill epithelium, as well as sodium–potassium ATPase (NKA) activity in osmoregulatory organs such as the gill, intestine, and kidney [14].

NKA, a universal membrane-bound enzyme, drives water and ion transport in various teleost osmoregulatory organs [15], mainly located in the mitochondria-rich cells of the gill filament [9,16]. Ion and water balance regulation is a critical physiological process for most fish. Freshwater (FW) fish face excessive hydration and ion loss through their gills and body surface. Fish can adapt to a hypotonic environment by increasing ion absorption and producing dilute urine [17]. When a euryhaline teleost makes the transition from hypoosmotic (FW) to hyperosmotic (SW) media, the fish tends to gain ions while losing water [15]. The gill *nka* mRNA plays an important role in acclimation during FW to SW transfer in teleosts, including rainbow trout, *Oncorhynchus mykiss* [18] and sea bream, *Sparus aurata* [19], and during SW to FW transfer in the black porgy, *Acanthopagrus schlegelii* [20].

Growth hormone (*gh*), another traditional SW-adapting hormone in teleosts, promotes animal survival in SW environments by antagonizing the actions of *prl* [21,22]. In salmonids, the osmoregulatory actions of *gh* are well established, particularly in terms of promoting SW tolerance [23]. Hormones of the *gh*/*prl* family must interact with transmembrane receptors such as *ghr*/*prlr* that initiate the JAK/STAT signaling pathway in order to affect target tissues during salinity/osmoregulatory stress [24]. The receptors of *gh*/*prl*/cortisol have been found in tilapia gills and kidneys using a variety of methods, including hormone binding studies [25,26], molecular cloning [27], immunolocalization [28], and in situ hybridization [9]. 

*prl* has been shown to regulate a variety of functions in teleost fishes, including reproduction, immunomodulation, pigmentation, and seasonal acclimatization [29,30]. Due to its significance, *prl* is generally perceived as a “freshwater adapting hormone”, required for maintaining hydromineral balance in several teleosts that live in FW [31,32,33,34]. In addition, *prl* release also increases in response to physiologically relevant reductions in extracellular osmolality [35]. *prl* regulates the hydromineral balance in euryhaline fish by acting on the gill, kidney, and intestine via *prlr* [36,37] to promote ion uptake and water-extruding processes [23,31,38]. *prlr* transcripts were most abundant in the kidney, fewer in the gill, intestine, brain, and spleen, and at very low levels in the pituitary and other tissues studied in turbot, *Scophthalmus maximus* [39]. *prlr* transcripts have been found to be expressed in the gills of tilapia, *Oreochromis niloticus* and rainbow trout, *Oncorhynchus mykiss* [40], goldfish, *Carassius auratus* [41], Japanese flounder, *Paralichthys olivaceus* [42], sea bream, *S. aurata* [43,44], pufferfish, *Takifugu rubripes* [45], tilapia, *O. mossambicus* [46,47], and zebrafish, *Danio rerio* [48].

Since black porgy, *A. schlegelii*, is an euryhaline teleost that migrates from near-shore shallow areas to coastal waters near land or in estuaries during its larval to the juvenile stage [49], osmoregulation is an important way for it to maintain a stable internal environment. The purpose of this study was to investigate the changes in *gh*/*prl* hormone gene expression in the pituitary gland, as well as their *ghr*/*prlr*/*gr* (isoform-1) receptors in the gill, kidney, and intestine, as well as serum osmolality and chloride concentrations when the black porgy were transferred from SW to 4 ppt and FW and vice versa at different salinity levels. 

## 2. Results

### 2.1. Serum Osmolality and Chloride Concentrations

**Figure 1:**
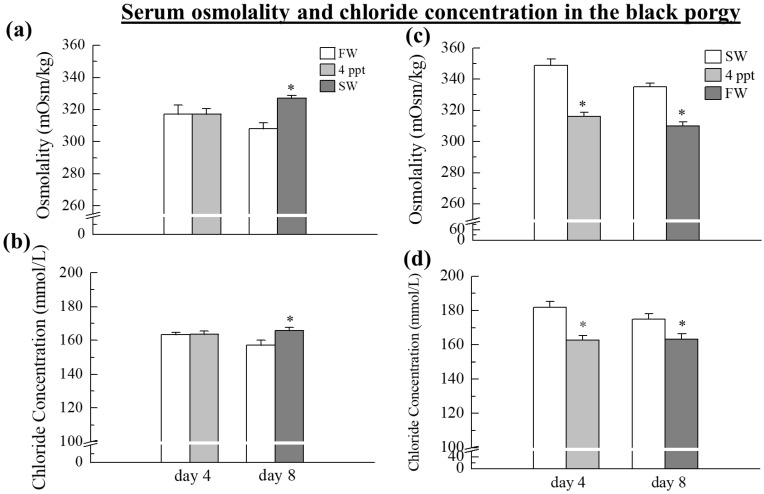
Analysis of the serum osmolality (**a**,**c**) and chloride concentrations (**b**,**d**) in black porgy on day 4 and 8 (n = 9, in each group). The results are expressed as the mean ± SEM (standard error of the mean). Asterisks (*) indicate significant differences (*p* < 0.05) between control and treated groups.

Both serum osmolality and chloride concentrations were significantly (*p* < 0.05) increased when the fish were transferred from FW to SW on day 8 (Figure 1a, b). On the other hand, during the transfer of fish from SW to 4 ppt and SW to FW, both serum osmolality and chloride concentrations were significantly (*p* < 0.05) reduced on day 4 and day 8 as compared to the SW group (Figure 1c,d). There was no significant difference in serum osmolality and chloride concentrations during the transfer from FW to 4 ppt on day 4 (Figure 1a,b). 

### 2.2. The Expression of prl and gh Transcripts in the Pituitary

**Figure 2:**
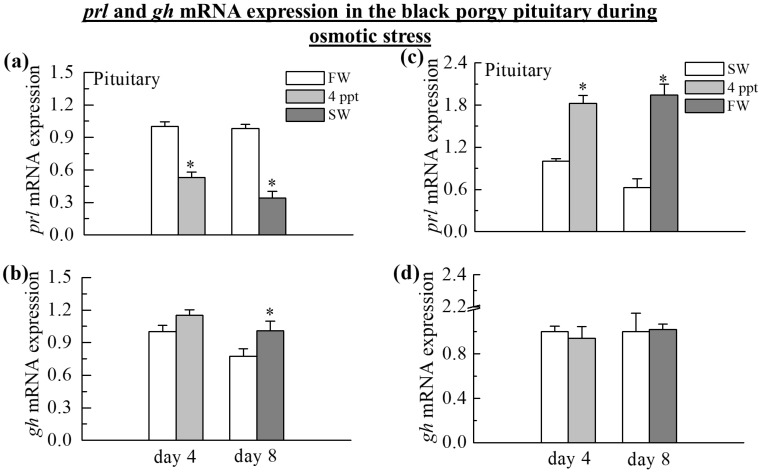
Q-PCR was performed to analyze the transcripts of *prl* (**a**) and *gh* (**b**) in the pituitary during the transfer of black porgy from FW to 4 ppt and SW, and (**c**,**d**) SW to 4 ppt and FW on day 4, and day 8. Gene expression was normalized to the control value on day 4. The results (n = 9) are expressed as the mean ± SEM. Asterisks (*) indicate significant differences (*p* < 0.05) between the control and treated group.

The quantitative real-time PCR (Q-PCR) analysis showed that the *prl* mRNA expressions were significantly reduced when the fish were transferred from FW to 4 ppt and SW on day 4 (0.5-fold, *p* < 0.05) and day 8 (2.8-fold, *p* < 0.05), respectively (Figure 2a). On the other hand, the *prl* transcripts were notably increased when the transfer was from SW to 4 ppt on day 4 (1.8-fold, *p* < 0.05) and from SW to FW on day 8 (3.1-fold, *p* < 0.05) (Figure 2c). Exposure to SW increased *gh* mRNA in the pituitary on day 8 at 34 ppt (1.3-fold, *p* < 0.05) in black porgy (Figure 2b). There was no significant difference in *gh* mRNA expression in the pituitary during the transfer from FW to 4 ppt on day 4 (Figure 2b) and from SW to 4 ppt on day 4 and FW on day 8 (Figure 2d).

### 2.3. The Expression of α-nka, prlr, gr, and ghr Transcripts in the Gill 

**Figure 3:**
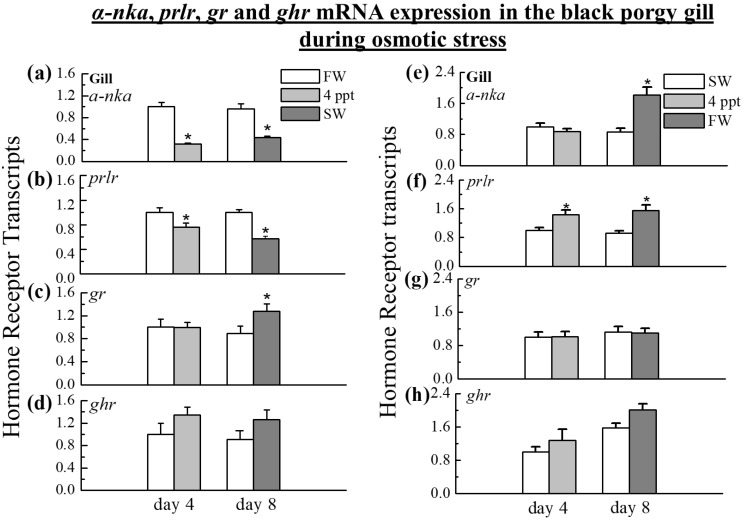
Q-PCR was performed to analyze the transcripts of *α-nka*, *prlr*, *gr*, and *ghr* in the gill during the transfer of black porgy (**a**–**d**) from FW to 4 ppt and SW, and (**e**–**h**) from SW to 4 ppt and FW on day 4, and day 8. The relative gene expression was normalized to the control (FW or SW group) value on day 4. The results (n = 9) are expressed as the mean ± SEM. Asterisks (*) indicate significant differences (*p* < 0.05) between the control and treated group.

The *α-nka* (0.32-fold in 4 ppt, *p* < 0.05; 0.41-fold in SW, *p* < 0.05) and *prlr* (0.75-fold in 4 ppt, *p* < 0.05 and 0.52-fold in SW, *p* < 0.05) transcripts were similarly decreased during the transfer from FW to 4 ppt on day 4 and SW on day 8 (Figure 3a,b). Interestingly, the SW acclimation significantly raised the GR transcripts (1.44-fold in SW, *p* < 0.05) on day 8 (Figure 3c), but no difference was found during the transfer from FW to 4 ppt on day 4 (Figure 3c). Similarly, the *ghr* transcripts were unaffected by increasing the salinity on day 4 and day 8 (Figure 3d). On the other hand, gill *prlr* transcripts were increased during the transfer from SW to 4 ppt on day 4 (1.44-fold in 4 ppt, *p* < 0.05) and from SW to FW on day 8 (1.70-fold in FW, *p* < 0.05) (Figure 3f). *α-nka*mRNA was increased in fish gills from SW to FW on day 8 (2.11-fold in FW, *p* < 0.05) but did not increase in fish from SW to 4 ppt on day 4 (Figure 3e). mRNA expression of *gr* and *ghr* in the gill was not changed by the osmoregulatory stress from SW to 4 ppt on day 4 (Figure 3g,h) and FW on day 8 (Figure 3g,h).

### 2.4. The Expression of α-nka, prlr, gr, and ghr in the Intestine 

**Figure 4:**
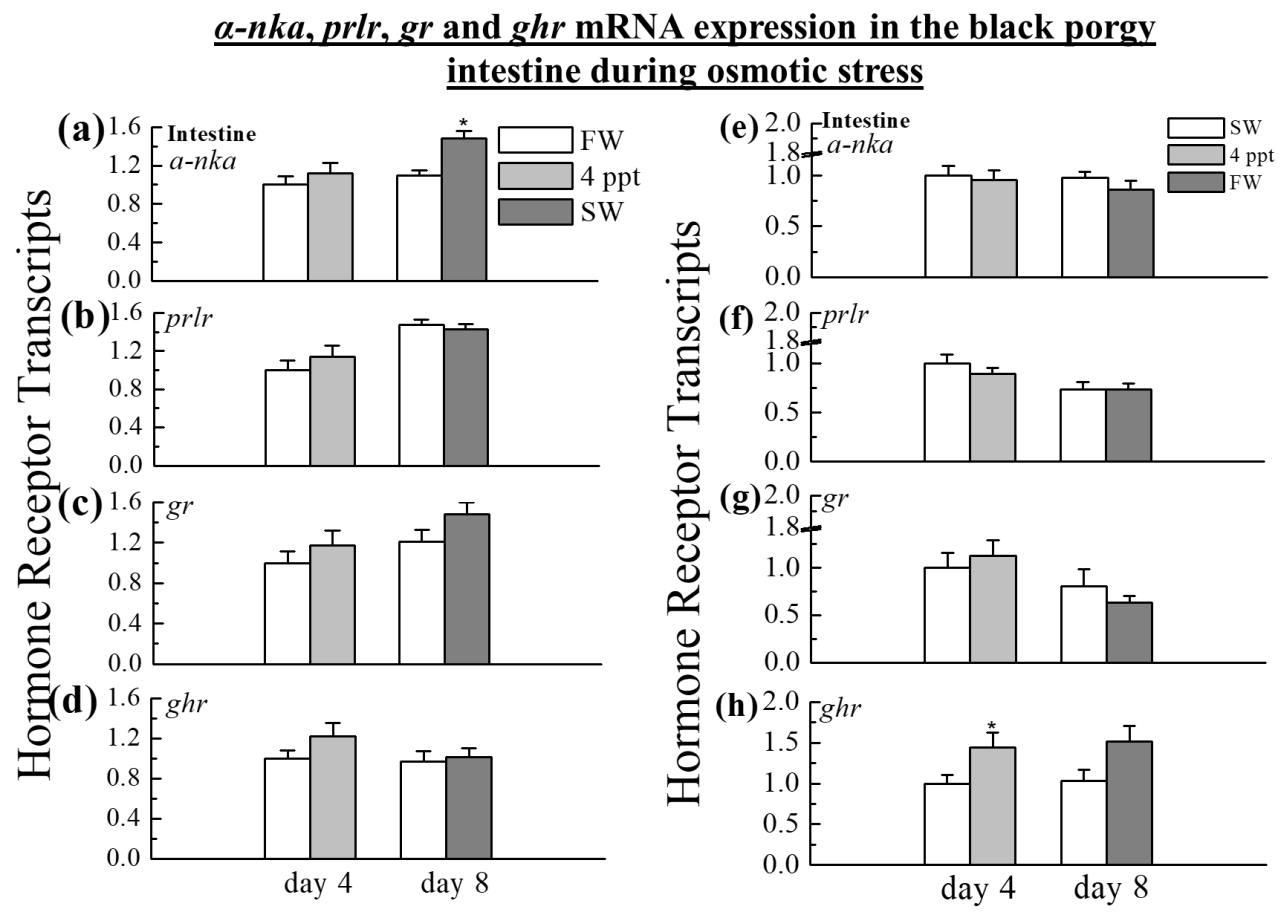
Q-PCR was performed to analyze the transcripts of *α-nka*, *prlr*, *gr,* and *ghr* in the intestine during the transfer of black porgy (**a**–**d**) from FW to 4 ppt and SW, and (**e**–**h**) from SW to 4 ppt and FW on day 4 and day 8. The relative gene expression was normalized to the control (FW or SW group) value on day 4. The results (n = 9) are expressed as the mean ± SEM. Asterisks (*) indicate significant differences (*p* < 0.05) between the control and treated groups.

The *α-nka* transcripts increased significantly during SW transfer on day 8 (1.3-fold in SW, *p* < 0.05) in the intestine compared to FW (Figure 4a). No significant changes in the expression levels were detected in the intestine on day 4 during the transfer from FW to 4 ppt (Figure 4a). Similarly, during the transfer from FW to 4 ppt and SW, there was no significant difference in the expression of *prlr*, *gr*, and *ghr* mRNA (Figure 4b–d). The *ghr* transcripts were increased in the intestine during the transfer from SW to 4 ppt (Figure 4h) on day 4 (1.44-fold in 4 ppt, *p* < 0.05), whereas on day 8, there was no difference in SW to FW transfer (Figure 4h). In the black porgy intestine, *α-nka*, *prlr*, and *gr* transcripts on day 4 and day 8 were not affected by the osmotic stress from SW to 4 ppt and FW (Figure 4e–g).

### 2.5. The Expression of α-nka, prlr, gr, and ghr in the Kidney

**Figure 5:**
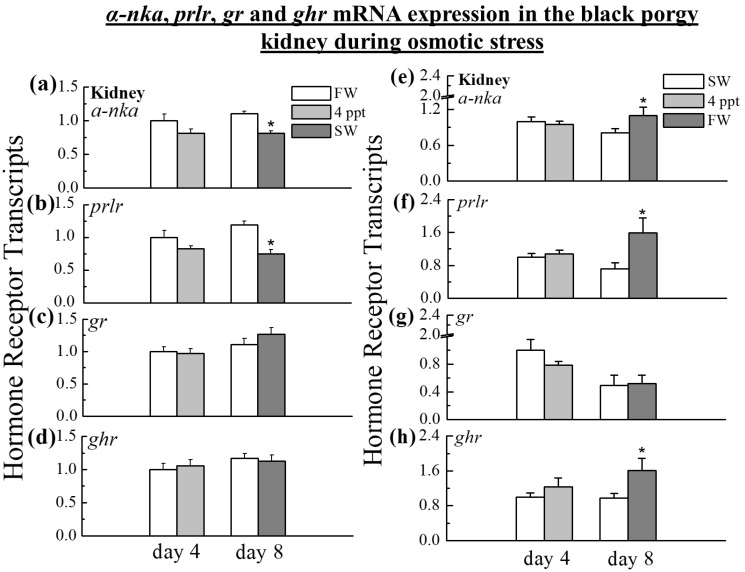
Q-PCR was performed to analyze the transcripts of *α-nka*, *prlr*, *gr*, and *ghr* in the kidney during the transfer of black porgy from FW to 4 ppt and SW (**a**–**d**), and from SW to 4 ppt and FW (**e**–**h**) on day 4 and day 8. The relative gene expression was normalized to the control value on day 4. The results (n = 9) are expressed as the mean ± SEM. Asterisks (*) indicate significant differences (*p* < 0.05) between the control and treated group.

During the transfer from FW to SW, the *α-nka* (0.81-fold in SW, *p* < 0.05) and *prlr* (0.62-fold in SW, *p* < 0.05) transcripts were significantly decreased in the kidney on day 8 (Figure 5a,b). On the other hand, the *α-nka* (1.3-fold in FW, *p* < 0.05), *prlr* (2.2-fold in FW, *p* < 0.05), and *ghr* (1.66-fold in FW, *p* < 0.05) transcripts were elevated during the transfer of fish from SW to FW on day 8 (Figure 5e,f,h). When the black porgy were transferred from FW to 4 ppt and SW, the *gr* and *ghr* mRNA showed no significant difference in the kidney (Figure 5c,d), and during the transfer from SW to 4 ppt and FW, fish did not show a significant difference in *gr* mRNA levels in the kidney on day 4 and day 8 (Figure 5g).

## 3. Discussion

The current study was carried out to investigate the expression pattern of hormones such as *prl*, *gh*, *prlr*, *ghr*, *gr*, and *α-nka* that are responsible for salinity and osmoregulatory stress in a euryhaline teleost, black porgy, during the transition from FW to 4 ppt and SW, and from SW to 4 ppt and FW acclimation at different time intervals. On day 4 and day 8, when the fish were transferred from SW to 4ppt and FW, their serum osmolality and chloride concentrations were significantly lower. On the other hand, these osmolality and chloride concentrations increased significantly on day 8, after transferring fish from 4 ppt to SW. When compared to FW-maintained fish, SW acclimation significantly increased serum osmolality and chloride levels in black porgy. As in other teleosts, serum chloride concentrations and osmolality decreased following transfer to FW [50,51,52]. During salinity changes, euryhaline teleosts face significant osmotic challenges. The endocrine system mediates osmoregulatory responses to salinity changes in the environment, which further initiates osmoregulatory tissues to modulate ion transport. Nevertheless, the gill is regarded as the primary site of total current sodium and chloride transport, which is controlled and mediated by chloride cell activity [53].

The expression of *gh* transcripts in osmoregulation varies among species [23]. The unaffected pituitary *gh* mRNA levels in SW and FW were reported in the euryhaline Mozambique tilapia [53,54,55], sea bass, *Dicentrarchus labrax* [56], and olive flounder, *Paralichthys olivaceus* [17]. Similarly, there was no significant difference in the mRNA expression of *gh* in the pituitary and *ghr* in the gill during FW to 4 ppt and SW to 4 ppt and FW transfer of black porgy at any time point in this study. However, the long-time exposure to the high salinity significantly increased the *gh* mRNA in the pituitary on day 8 during the transfer of black porgy from 0 ppt to 34 ppt. The lack of change in the *gh* levels in black porgy does not rule out a possible role in osmoregulation in this species. On day 4, kidney *ghr* mRNA expression was also not changed in the response to salinity and osmoregulatory stress. Long-term exposure of SW to FW resulted in the changes in *ghr* transcripts in the kidney of the black porgy on day 8. The FW transfer induced the increases in *ghr* gene expression in the black porgy kidney on day 8. Continuous *ghr* mRNA expression in the kidney from 6 h after transfer to the end of the experiment indicated the possibility of a centralized *gh*/*Igf* axis action, as previously described in salmon [57].

Previous research in teleosts indicates that the pituitary hormone *prl* regulates salt and water homeostasis by influencing ion retention and water uptake via peripheral osmoregulatory organs [39]. Consistently, the present study revealed that *prl* mRNA transcripts increased from SW to 4 ppt and FW transfer on day 4 and day 8, and decreased significantly from FW to 0 ppt and SW transfer on day 4 and day 8 in the pituitary. The pituitary *prl* contributes to the fish systemic response to salinity changes [58]. *prl* regulates ion-conserving and water-secreting processes in osmoregulatory tissues such as the gills, kidney, intestine, and urinary bladder to mediate FW acclimation [48]. *prl* gene expression rises in response to the decreases in environmental salinity [59,60]; in some cases, these rises are triggered by direct extracellular osmolality [61]. A decrease in *prl* mRNA levels was shown in black chin tilapia beginning on the first post-experimental day after SW exposure and lasting throughout the SW period, which was restored in FW to baseline control levels [62]. This is consistent with the observation made in isolated *prl* cells that fish salinity acclimation history influences osmotic responsiveness [63,64,65,66,67]. Based on the wide range of tissues known to respond to *prl* across teleosts, it is widely assumed that *prl* is a conserved regulator of physiological responses to low-salinity environments [23]. 

As with the *prl* transcripts in the pituitary, a similar pattern of *prlr* mRNA expressions was observed in the gill when the black porgy transferred from SW to 4 ppt and FW, and from FW to 4 ppt and SW. The increased mRNA expression of *prl* in the pituitary and *prlr* in the gill of FW black porgy suggests that *prl* and *prlr* may have a potential function in FW adaptation. At 12 and 24 h, gill *prlr* mRNA levels were lower in SW-transferred fish compared to FW controls. Despite previous research showing that *prlr* gene expression is reduced in long-term SW-acclimated tilapia [46], this study concludes that rapid down-regulation of *prlr* occurs with SW acclimation [53]. It should also be stated that other factors, such as extracellular osmolality, may play a role in the regulation of *prlr* expression in teleost gills [53]. The concurrent changes in pituitary *prl* and gill *prlr* mRNA levels observed during the transfer of fish from SW to 4 ppt and FW on day 4 and 8 in this study support a role for *prl* in promoting FW acclimation in black porgy.

In the present study, increased *prlr* and *α-nka* transcripts in the gill were associated with the transfer of fish from high salinity to low salinity. The expression of *α-nka* mRNA in the intestine increased when the fish were transferred from FW to SW on day 8. When the black porgy were transferred from SW to FW, the transcripts of *prlr*, *α-nka*, and *ghr* were significantly increased in the kidney. Branchial *α-nka* mRNA has been reported to be higher in FW in black porgy [68], which is consistent with the current study’s findings that NKA levels increased on day 8 in FW compared to SW. The expression of the *α-nka* gene in the intestine of the euryhaline black porgy increases during the transition from FW to SW on day 8, demonstrating the importance of this enzyme in successful marine osmoregulation [69]. Euryhaline fishes can maintain a narrow range of internal osmolality in response to a wide range of environmental salinities. Fish acclimated to FW experience hypoosmotic conditions with osmotic water gain and diffusive ion loss, whereas fish accustomed to SW experience dehydration. Teleost fishes have evolved complex physiological functions at the level of osmoregulatory organs such as the gill, kidney, and intestine, which are primarily governed by the endocrine system, to deal with such challenges [33,70,71]. 

The SW adaptation increased gill *gr* expression within 24 h and maintained it for up to 4 days in SW, indicating the importance of osmoregulation in a hyperosmotic environment [9]. In the gill of rainbow trout, the *gr* mRNA expression was significantly increased during acute netting stress [72] and SW acclimation [72,73]. On the other hand, the *gr* mRNA expressions of black porgy were unaffected by salinity and osmoregulatory stress on day 4 at 4 ppt. When the fish were transferred from FW to SW, the *gr* transcripts in the gills were significantly increased on day 8, implying that the *gr* transcript played a significant role in black porgy to cope with the homeostasis during SW acclimation. The intestine of fish, as with the gills, is an important osmoregulatory organ. Cortisol is a key regulator of fish osmoregulation, promoting intestinal ion and water absorption [74,75,76], and the SW adaptation of euryhaline teleosts, with a significant increase in *gr* mRNA in the intestine [77,78]. *gr* transcripts were significantly increased in the intestine and kidney of the SW fish on day 1 in tilapia [9], whereas neither kidney nor intestinal gene mRNA expression of *gr* changed during the course of salinity and osmoregulatory stress on day 4 and day 8. The data revealed the differences in the regulation and expression of the *gr* among organs such as the kidney, gill, and intestine that have different functions [6,79,80].

Comparative approaches of the present study concluded that the transfer of black porgy from SW to 4 ppt and FW, and from FW to 4 ppt and SW acclimation showed the opposite expression in the *prl* in the pituitary and *prlr* in the gill of black porgy. *α-nka* transcripts were increased in the fish gill acclimated to FW as compared to SW. The current study findings showed that the gill *gr* transcript was solely employed to reconcile homeostasis in the black porgy under salinity stress. Overall, during the transfer of black porgy from FW to 4 ppt and SW to 4 ppt, neither the kidney nor intestinal gene mRNA expression of *prlr*, *α-nka*, *gr*, and *ghr* changed during the course of the transfer from FW to 4 ppt and from SW to 4 ppt on day 4. Long-term salinity and osmoregulatory stress exposure, i.e., day 8, only affects the above transcripts in the black porgy intestine and kidney. The delayed increase in *prlr*, *gr*, *α-nka*, and *ghr* might be caused by the initiation of the endocrine process depending on the environmental salinity as well as the exposure time intervals among the various osmoregulatory organs of the black porgy. 

## 4. Materials and Methods

### 4.1. Experimental Fish

Black porgy (2-year-old, n = 90; body weight = 375.70 ± 13.65 g, body length = 27.90 ± 0.30 cm) were cultured in SW and natural light system at a university aquarium (water temperature ranged from 19 to 24 °C). Fish were given pelleted dry food ad libitum at 1% (*w*/*v*) of their estimated body weight per day. The fish were anesthetized with 1% (*v*/*v*) glycophenol monophenyl ether and decapitated for each experiment. Pituitary, gill, kidney, and intestine samples were collected and stored in liquid nitrogen at −80 °C. The current experiments were carried out in accordance with the principles and procedures established by the Institutional Animal Care and Use Committee at National Taiwan Ocean University in Taiwan.

### 4.2. Experiment of Osmotic Transfer

In this study, two salinity experiments were carried out. Fish (n = 9 per group) were kept at 33 ppt for the experiment. The fish were randomly divided into two groups and kept in either SW or FW. After a 30-day acclimation period, the water salinity of fish (n = 9 per group) kept in FW was gradually changed to 4 ppt within 4 days (treated group; 4 day group, n = 9) and then from 4 ppt to 34 ppt SW) within another 4 days (treated group; 8 day group, n = 9). Similarly, fish kept in SW (n = 9 per group) were gradually changed to 4 ppt within 4 days (treated group; 4 day group, n = 9) and then from 4 ppt to FW within another 4 days (treated group; 8 day group, n = 9). Respective control groups (n = 9 per group) received the transfer but no salinity changes. The fish were sampled four and eight days after salinity transfer. Every day, the salinity of the water was checked and corrected as needed. The fish were anesthetized in 2-phenoxyethanol (0.4 mL/L) at the end of the experiment, and blood was collected from caudal vessels with a syringe in less than 5 min. The fish were then decapitated, and samples of the gills, intestine, kidney, and pituitary gland were taken, snap-frozen in liquid nitrogen, and stored at −80 °C until RNA extraction. Serum was obtained by centrifugation at 1000× *g* for 5 min at 4 °C and stored at −20 °C until further analysis.

### 4.3. RNA Extraction, the First-Strand cDNA Synthesis, and Cloning

TRIzol^®^ reagent (Gibco BRL; Grand Island, NY, USA) was used to isolate RNA from the pituitary, gill, intestine, and kidney, which was then reverse transcribed according to the manufacturer’s protocol. The resulting cDNA was used as a template for subsequent PCR amplification of the genes used in this study.

The *prl* (accession number EU 165342), *prlr* (EF 467927), *α-nka* (EF 621407), *ghr* (AF 502071), and *gr* (AY 921612) genes were cloned from the black porgy gill cDNA. Multiple alignments of previously published sequences of the respective genes were constructed using the CLUSTAL X program (Modified version 1.81, 10-06-2010) to identify the conserved region; primers were then designed based on this information (Table 1). PCR reactions were performed with 2.5 µL of 10X reaction buffer (200 mM Tris-HCl (pH 8.4), 500 mM KCl), 1 μL of 10 mM dNTP, 1 µL of 2 mM MgCl2 and 0.5 μL each of 10 µM sense and antisense primers, 1 μL cDNA and 0.2 μL superscript enzyme (Invitrogen; Calsbad, CA, USA) in a final volume of 25 µL. The PCR conditions were set as follows: 94 °C for 5 min, 94 °C for 30 s, 50 °C for 30 s, 72 °C for 30 s for 35 cycles, and 72 °C for 10 min. The PCR products were validated using 1.5% (*w*/*v*) agarose gel electrophoresis and ethidium bromide staining, and DNA fragments were excised using a Gel-M TM Gel Extraction system Kit (Bio 101) (VIOGENE; La Jolla, CA, USA) and cloned into pGEM^®^ -T Easy vector (Promega; Madison, WI, USA). The insert was sequenced on a plasmid using a dye terminator cycle sequencing kit (Perkin Elmer; Foster City, CA, USA) and submitted to BLAST for comparison to known sequences in the NCBI database.

### 4.4. Quantification of α-nka, prl, prlr, gr, and ghr by Q-PCR Analysis

Using the iQ^TM^ Multicolor Real-Time PCR Detection system (Bio-Rad Co., Hercules, CA, USA), Q-PCR was used to examine the gene expression of *α-nka*, *prl*, *prlr*, *ghr*, and *gr* in the pituitary, gill, kidney, and intestine during FW and SW acclimation. Primer expression software (Version 3.0, 30-06-2008, Applied Biosystem; Foster City, CA, USA) was used to design the primers (Table 1). According to our previous study, gene quantification of standards, samples, and controls was performed simultaneously in a Q-PCR machine (iQTM Multicolor Real-Time PCR Detection System; Bio-Rad Co.) using iQTM SYBR green (Bio-Rad Co.) as a dsDNA minor-groove binding agent, forward and reverse primers, and water [81]. The slope of the relationship between log input cDNA (transcript concentrations) vs. Ct was used to calculate PCR efficiency. The relative expression levels of *α-nka*, *prl*, *prlr*, *ghr*, and *gr* in the pituitary, gill, intestine, and kidney were calculated. The control data of the SW or FW group on day 4 were calculated as 1 and then calibrated with other data in the same experiment. The standard curve correlation was −0.999.

### 4.5. Statistical Analysis

Data are presented as means standard deviation of the mean (mean ± SEM). The Student *t*-test was also used to determine whether there were significant differences (*p* < 0.05) between the control and treated group, which are denoted by asterisks (*).

## Figures and Tables

**Table 1:** Specific primers used for Q-PCR analysis. F: forward primer, R: reverse primer.

Gene	Orientation	Nucleotide Sequences	Usage
*prl*	F	5′-AGTCGGACCTGTTGTCATTGG-3′	Q-PCR
	R	5′-AGGCTGTTGGCAGAGTTGGA-3′	Q-PCR
*prlr*	F	5′-CAACGCGCTGGGAAGAAC-3′	Q-PCR
	R	5′-AGGATTGGGCTTGACGATGTAC-3′	Q-PCR
*gh*	F	5′-CCTGCAGGATTTCTGTAACTCTGAT-3′	Q-PCR
	R	5′-TGGGCTGCGCTGTGTCT-3′	Q-PCR
*ghr*	F	5′-CCGTCAGCTTTCCTGATGATG-3	Q-PCR
	R	5′-TGTCCTGGTCGTGGAGATCTG-3′	Q-PCR
*gr*	F	5′-AACACAGATGGCCAGCACAAC-3′	Q-PCR
	R	5′-CTTCCTTCGGATCTTATCGATGAT-3′	Q-PCR
*α-nka*	F	5′-ACCGTGGCCCACATGTG-3′	Q-PCR
	R	5′-GGTCCCGCTCTGGTTCTCA-3′	Q-PCR

## Data Availability

Data are contained within this article. Raw data are available upon request from the corresponding authors.
